# Induction of somatopause in adult mice compromises bone morphology and exacerbates bone loss during aging

**DOI:** 10.1111/acel.13505

**Published:** 2021-11-23

**Authors:** Manisha Dixit, Silvana Duran‐Ortiz, Godze Yildirim, Sher Bahadur Poudel, Leeann D. Louis, Andrzej Bartke, Mitchell B. Schaffler, John J. Kopchick, Shoshana Yakar

**Affiliations:** ^1^ David B. Kriser Dental Center Department of Molecular Pathobiology New York University College of Dentistry New York New York NY USA; ^2^ Edison Biotechnology Institute and Dept. of Biomedical Sciences Ohio University Athens OH USA; ^3^ Department of Biomedical Engineering City College of New York New York NY USA; ^4^ Southern Illinois University School of Medicine Springfield IL USA

**Keywords:** bone, growth hormone, micro‐CT, Raman microspectroscopy, sexual dimorphism

## Abstract

Somatopause refers to the gradual declines in growth hormone (GH) and insulin‐like growth factor‐1 throughout aging. To define how induced somatopause affects skeletal integrity, we used an inducible GH receptor knockout (iGHRKO) mouse model. Somatopause, induced globally at 6 months of age, resulted in significantly more slender bones in both male and female iGHRKO mice. In males, induced somatopause was associated with progressive expansion of the marrow cavity leading to significant thinning of the cortices, which compromised bone strength. We report progressive declines in osteocyte lacunar number, and increases in lacunar volume, in iGHRKO males, and reductions in lacunar number accompanied by ~20% loss of overall canalicular connectivity in iGHRKO females by 30 months of age. Induced somatopause did not affect mineral/matrix ratio assessed by Raman microspectroscopy. We found significant increases in bone marrow adiposity and high levels of sclerostin, a negative regulator of bone formation in iGHRKO mice. Surprisingly, however, despite compromised bone morphology, osteocyte senescence was reduced in the iGHRKO mice. In this study, we avoided the confounded effects of constitutive deficiency in the GH/IGF‐1 axis on the skeleton during growth, and specifically dissected its effects on the aging skeleton. We show here, for the first time, that induced somatopause compromises bone morphology and the bone marrow environment.

## Graphical abstract

Induced somatopause during aging causes a drop in GH/IGF‐1 signaling, inhibits radial bone expansion and associates with increased bone marrow adiposity. At the bone tissue level, induced somatopause leads to progressive declines in osteocyte lacunar density and connectivity.

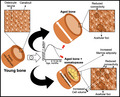

**Abbreviations**
ALS: acid labile subunit
B.Ar: bone area
BMD: bone mineral density
BV/TV: bone volume/total volume
cGHRKO: constitutive GHRKO
Cn.N: canaliculi number
Ct.Th: cortical bone thickness
DMP‐1: dentin matrix protein‐1
FGF23: fibroblast growth factor‐23
GH: growth hormone
GHR: GH receptor
IGF‐1: insulin‐like growth factor‐1
IGFBP: IGF‐binding protein
iGHRKO: inducible global‐GHR knockout
IHC: immunohistochemistry
IL6: interleukin‐6
iLID mouse model: inducible liver‐IGF‐1 deleted
J_0_
: polar moment of inertia
Lac.N: lacunae number
Lac.V: lacunae volume
LCN: lacunar‐canalicular network
M.Ar: marrow area
mCT: micro computed tomography
RCA: relative cortical bone area
SOST: sclerostin
T.Ar: total cross‐sectional area
Tb.N: trabecular bone number
Tb.Th: trabecular bonethickness
TMD: tissue mineral density
TRAP: tartrate acid phosphatase

## 1 INTRODUCTION

Growth hormone (GH) and the insulin‐like growth factor‐1 (IGF‐1) are key endocrine factors regulating body composition, acquisition of peak bone mass, and maintenance of bone mineral density (BMD) through development and aging (Yakar et al., 2018). During aging the somatotropic signals of the GH/IGF‐1 axis decline, a state termed somatopause. Somatopause has been considered a significant cause for changes in body composition, BMD, as well as increased morbidity and mortality (Graham et al., 2009; Krysiak et al., 2009; Lanfranco et al., 2003). In humans, bone content of IGF‐1 and the IGF‐binding protein (IGFBP)‐5, which keeps the IGF‐1 pool in the bone matrix, decline by ~60% between the ages of 20 and 60 years (Nicolas et al., 1994) (Mohan & Baylink, 1997; Nicolas et al., 1995). *In vitro* studies have shown resistance to somatotropic stimulation in osteoblasts from old versus young subjects (Pfeilschifter et al., 1993). In addition, several clinical studies have correlated somatopause with decreases in BMD. The Framingham Osteoporosis Study of men and women aged 72–94 indicated that higher serum IGF‐1 levels were associated with greater BMD in very old women (Langlois et al., 1998). A few studies have shown that reductions in IGF‐1 levels were associated with increased fracture risk at several skeletal sites (Garnero et al., 2000; Szulc et al., 2004), while others did not find relationships between GH/IGF‐1 and BMD during aging (Kassem et al., 1994; Lloyd et al., 1996). GH replacement therapy in young or adult subjects with GH deficiency [GHD] showing reduced BMD and increased risk for fracture (Rosen et al., 1997; Wuster et al., 2001), effectively increased BMD (Baum et al., 1996; Janssen et al., 1998). Targeting somatopause in a randomized double‐blinded control trial showed that 3 years of GH treatment (of women aged 50–70) was beneficial for bone and fracture outcomes *even 10 years after treatment ceased* (Krantz et al., 2015). However, trials with rhGH or rhIGF‐1 in elderly patients are few and yielded conflicting results (Ghiron et al., 1995; Holloway et al., 1997; Marcus et al., 1990; Rudman et al., 1990).

Age‐associated somatopause in mice differs from that of humans; aged mice show decreases in GH peaks, but unlike humans, IGF‐1 levels remain relatively plateaued from 12 months onward (Yuan et al., 2009). Furthermore, mouse models with constitutive disruption of GH/IGF‐1 cannot clearly distinguish between developmental and age‐related effects of GH/IGF‐1 on the skeleton. To overcome this limitation, we and others have used an inducible liver‐IGF‐1‐deleted (iLID) mouse model to temporally reduce IGF‐1 during aging. An initial report by our group (Gong et al., 2014), and others (Ashpole et al., 2016a, 2016b) revealed that liver‐IGF‐1 deletion during aging prevented long bones from undergoing normal age‐related periosteal expansion, resulting in more narrow, that is, slender and mechanically inferior bones (Gong et al., 2014). However, the iLID mice show significant elevations in pituitary GH secretion, which has deleterious effects on overall health and secondary effects on bone (Gong et al., 2014). Thus, the iLID does not completely reproduce the loss of both the GH and IGF‐1 that drives somatopause in human.

**FIGURE 1:**
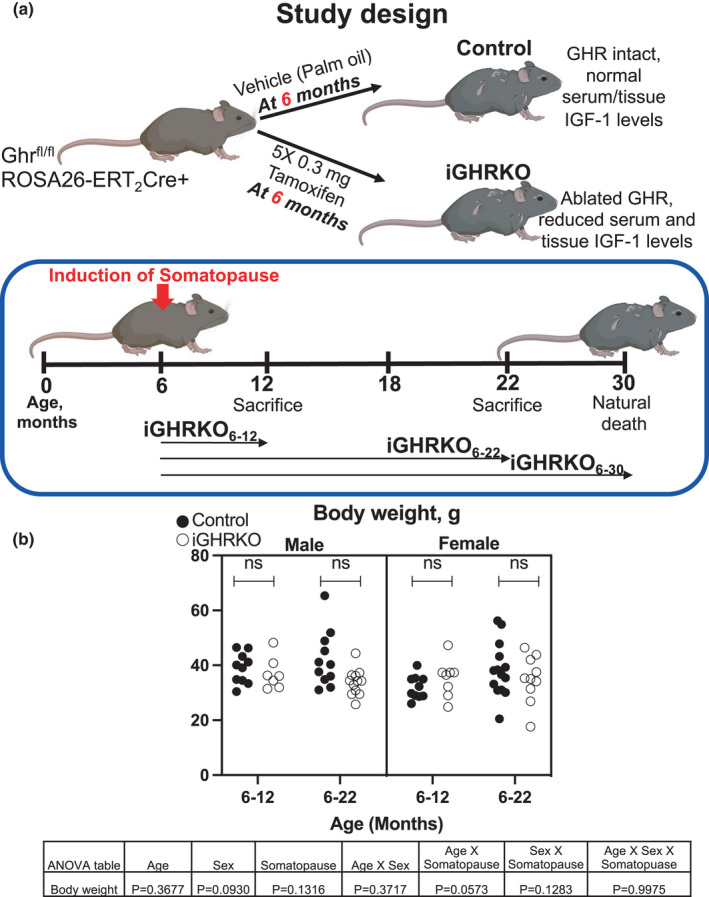
Schematics of the experimental design. (a) Mice were induced with somatopause at 6 months and bones were dissected at 12 (iGHRKO_6‐12_), 22 (iGHRKO_6‐22_), and 30 (iGHRKO_6‐30_) months of age. (b) Body weight taken at dissection. Data presented as mean ± SEM, tested by three‐way ANOVA, significance accepted at *p *< 0.05. Sample size: male Control_6‐12_=10, male iGHRKO_6‐12_=7, female Control_6‐12_=10, female iGHRKO_6‐12_=8, male Control_6‐22_=11, male iGHRKO_6‐22_=12, female Control_6‐22_=14, female iGHRKO_6‐22_=10

Here, we use a unique mouse model of age‐induced somatopause that reveals the significant roles played by GH/IGF‐1 in bone morphological and compositional characteristics during aging. We used the tamoxifen‐inducible ubiquitously expressed Cre mice in conjunction with the GHR‐floxed mice and generated an inducible global‐GHR knockout (iGHRKO, also known as 6mGHRKO) mice, a model previously validated (Junnila et al., 2016). Once injected with tamoxifen, iGHRKO mice show reductions in serum and tissue IGF‐1 and blockade of GH actions due to ablation of the GHR. Thus, the iGHRKO mice represent a good model for the human somatopause. We studied one cohort of mice in which somatopause was induced at 6 months and bones were dissected at 12 (iGHRKO_6‐12_), 22 (iGHRKO_6‐22_), and ~30 (iGHRKO_6‐30_) months of age (at the time of death) (Figure 1). Lifespan studies of this cohort showed sexual dimorphic effects where female iGHRKO mice exhibited increased lifespan without reduced pathology or changes in insulin resistance when compared to controls, while male iGHRKO mice did not show extended lifespan, but had decreases in markers of oxidative stress in peripheral tissues, and increased insulin sensitivity (Silvana et al., 2021). Here, we studied bone morphology by micro‐computed tomography (mCT), matrix composition by Raman spectroscopy, osteocyte connectivity by confocal microscopy imaging, and bone resorption/osteocyte senescence markers by immunohistochemistry. Our study revealed the fundamental roles played by the GH/IGF‐1 axis in regulating bone morphology and matrix composition during aging.

## 2 RESULTS

### 2.1 Induction of somatopause in adult mice resulted in sex‐dependent compromised skeletal morphology

**FIGURE 2:**
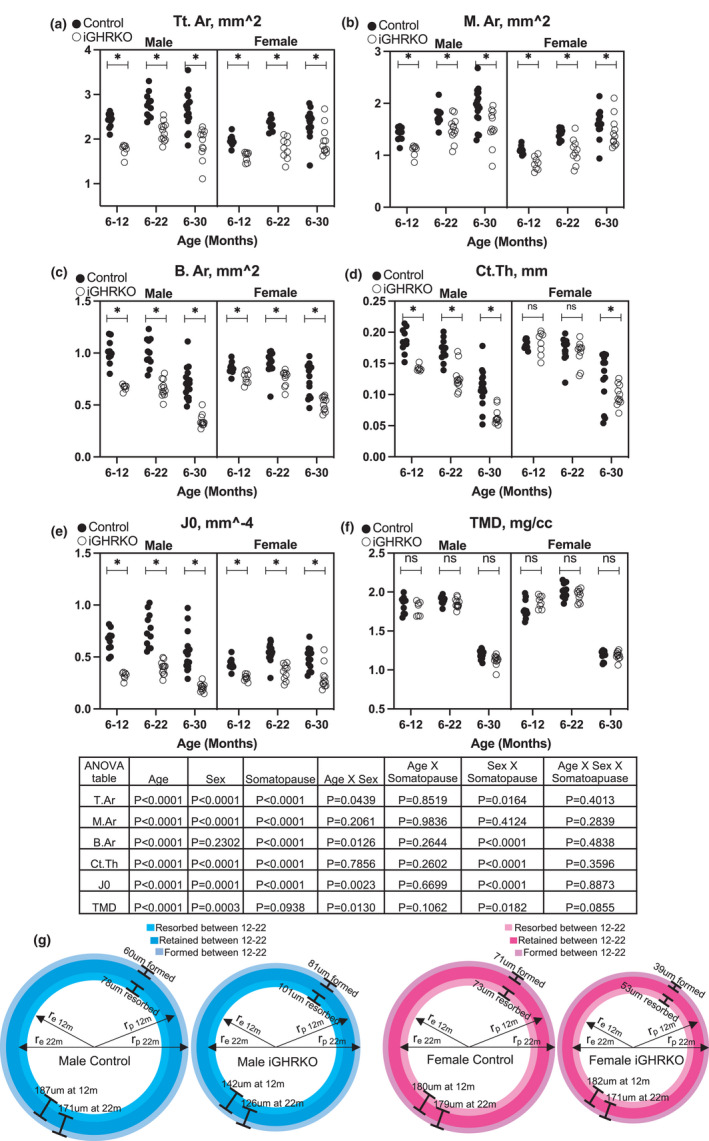
Femur morphology of somatopause‐induced mice resulted in failure of bones to undergo the normal radial expansion at the periosteal surfaces. mCT parameters were taken at the femur mid‐diaphysis of mice at the indicated ages. (a) Total cross‐sectional area (T. Ar), (b) marrow area (M. Ar), (c) bone area (B. Ar), and (d) cortical bone thickness (Ct. Th), (e) Polar moment of inertia (J_0_) and (f) bone tissue mineral density (TMD). (g) Sexual dimorphic effects of somatopause on bone remodeling during aging. The periosteal (outer) radius (r_p_) was calculated from T. Ar (r_periosteal_ = (T. Ar / π)^1/2^), and the endosteal (inner) radius (r_e_) from the M. Ar (r_inner_ = (M. Ar / π)^1/2^) of 12‐ and 24‐month‐old mice. Data presented as mean values of radii. Data presented as mean±SEM, tested by three‐way ANOVA, significance accepted at *p *< 0.05. Sample size: male Control_6‐12_=10, male iGHRKO_6‐12_=7, female Control_6‐12_=10, female iGHRKO_6‐12_=8, male Control_6‐22_=11, male iGHRKO_6‐22_=12, female Control_6‐22_=14, female iGHRKO_6‐22_=10, male Control_6‐30_=17, male iGHRKO_6‐30_=11, female Control_6‐30_=13, female iGHRKO_6‐30_=11

Micro‐CT (mCT) analyses revealed that induction of somatopause after peak bone acquisition (at 6 months of age) resulted in failure of bones to undergo the normal radial expansion at the periosteal surfaces. Male iGHRKO mice exhibited significantly less total cross‐sectional area (T. Ar) across all time points (~26%), suggesting that radial expansion was reduced due to lower periosteal bone apposition (Figure 2A). Accordingly, marrow area (M. Ar) was also lower (~19%), suggesting that endosteal resorption was reduced in iGHRKO male mice (Figure 2B). Bone area (B. Ar) and cortical bone thickness (Ct. Th) were lower in male iGHRKO mice (~40% and ~31%, respectively), and both contributed to lower polar moment of inertia (J_0_) (~54%) in iGHRKO male mice (Figure 2C‐E). Bones of female iGHRKO mice showed a similar phenotype to male iGHRKO mice, including lower T. Ar (~19%), B. Ar (~20%), M. Ar (~20%), and J_0_ (~35%) across all ages (Figure 2A‐E). However, reductions in Ct. Th in female iGHRKO mice were apparent only at 30 months, but not at 12 or 22 months of age, suggesting that periosteal bone formation and endosteal expansion were coupled in females until 22 months. Tissue mineral density (TMD), measured by mCT, at the mid‐diaphysis did not differ between iGHRKO mice and controls for males or females at any time point (Figure 2F). Both sexes showed a decline in BMD from 22 to 30 months, as is typically seen in aged mice (Boskey & Coleman, 2010).

The morphology data revealed a sexual dimorphic response to induced somatopause in the aging bone. We used an idealized cylindrical model for the mid‐diaphysis to summarize these differences (Figure 2G). The periosteal (outer) radius from T. Ar (r_periosteal_ = (T. Ar/π)^1/2^), and the endosteal (inner) radius from the M. Ar (r_endosteal_ = (M. Ar/ )^1/2^) were calculated from mCT data of 12‐ and 22‐month‐old mice. Differences in the periosteal radii indicated the average amount of periosteal formation, while differences in the endosteal radii indicated the average amount of endosteal bone resorption. Control males and females showed comparable amounts of periosteal bone formation and endosteal bone resorption. In contrast, male iGHRKO mice had increased periosteal apposition and endosteal loss, while female iGHRKO mice had reduced both processes (Figure 2G).

**FIGURE 3:**
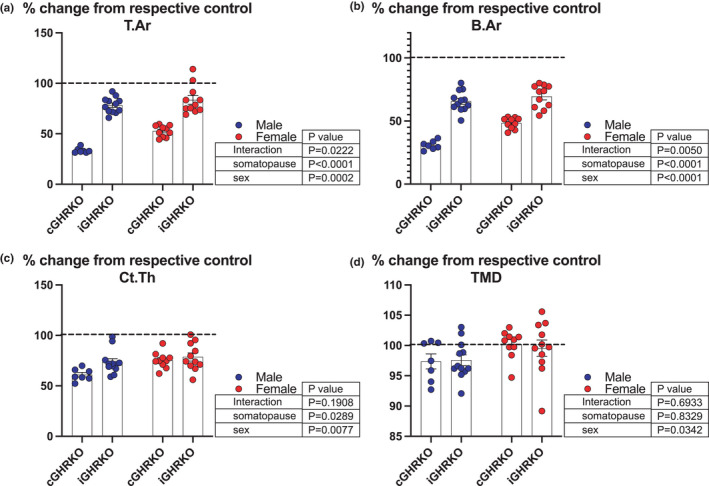
Bone morphology changes in response to constitutive GHRKO versus induced somatopause in the iGHRKO mice. mCT parameters were taken at the femur mid‐diaphysis of 30‐month‐old female and 22–24‐month‐old male mice. Values are presented as percent of controls of the same sex and age. Data presented as mean ± SEM, tested by two‐way ANOVA, significance accepted at *p *< 0.05. Sample size: Male GHRKO=7, male iGHRKO_6‐30_=12, female GHRKO=10, female iGHRKO_6‐30_=11

The magnitude of changes in morphological traits of cortical bone in response to induced somatopause (in the iGHRKO) was compared with those of constitutive GHRKO (cGHRKO) in male and female mice (Figure 3). As previously reported, T. Ar (Figure 3A) and B. Ar (Figure 3B) reduced by 50% in cGHRKO mice when compared to their cognate controls. In contrast, induction of somatopause during adulthood reduced these traits by only 25% in the iGHRKO mice. Surprisingly, however, Ct. Th (Figure 3C) reduced to a similar magnitude in both iGHRKO and cGHRKO mice as compared to their cognate controls. Thinning of the cortical envelope during aging in the iGHRKO mice to the same level as the cGHRKO has significant implications on bone strength. Cortical TMD was not affected by constitutive or induced somatopause (Figure 3D).

Trabecular bone volume (distal femoral metaphysis) declined with age (Figure S1A), and was principally associated with reductions in Tb.N by 22 months of age (Figure S1B). However, trabecular bone thickness (Figure S1C) was not affected. BMD reduced with age but did not differ between control and iGHRKO mice except in 22‐month‐old males (Figure S1D). However, female iGHRKO mice showed higher bone volume and trabecular number at 12 and 22 months as compared to their respective controls. Representative 3D volumes of the cortical bone at the femur mid‐diaphysis and trabecular bone at the distal metaphysis are shown in Figures S2–4.

### 2.2 Induction of somatopause in adult mice associates with changes in the connectivity of the osteocyte lacunar‐canalicular system

**FIGURE 4:**
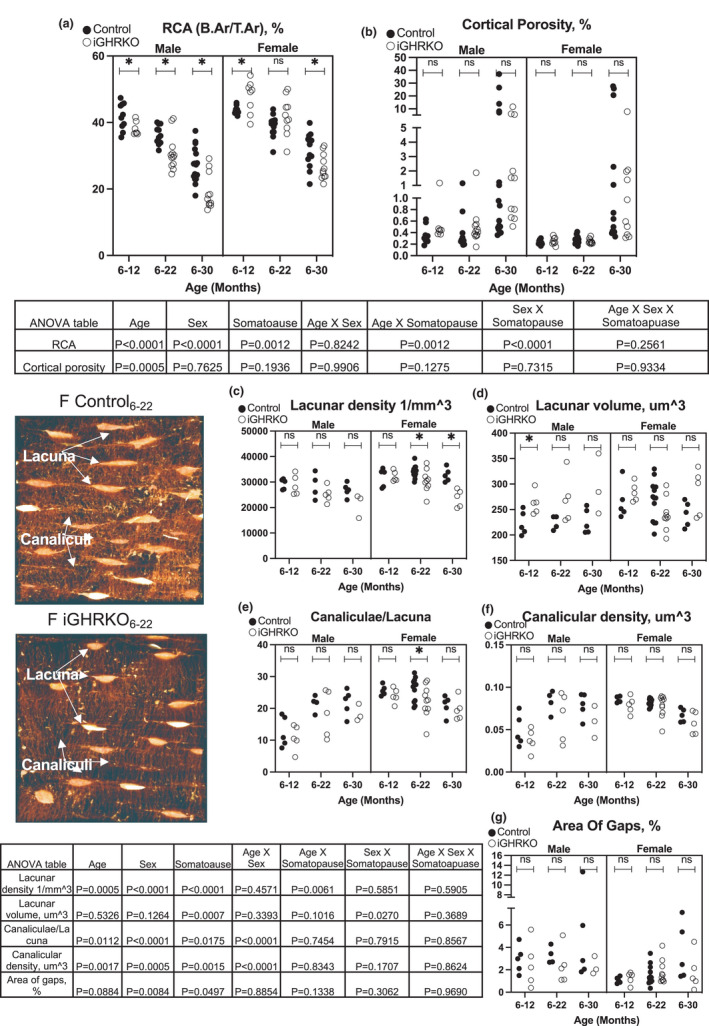
Somatopause showed sexual dimorphic effects on the LCN. (a) Relative cortical area (RCA) and (b) cortical bone porosity, calculated using mCT at the femur mid‐diaphysis. Sample size for A and B: male Control_6‐12_=10, male iGHRKO_6‐12_=7, female Control_6‐12_=10, female iGHRKO_6‐12_=8, male Control_6‐22_=11, male iGHRKO_6‐22_=12, female Control_6‐22_=14, female iGHRKO_6‐22_=10, male Control_6‐30_=17, male iGHRKO_6‐30_=11, female Control_6‐30_=13, female iGHRKO_6‐30_=11. (c) Lacunar density, the number of lacunae in a given volume of bone. (d) Lacunae volume became smaller with age (*p* < 0.001). Loss of canalicular connectivity, expressed as (e) the average number of canaliculi per lacuna and (f) the density of canaliculi per surface area of lacuna. (g) Area devoid of lacunae and canaliculi, assessed as a percentage of the total area. Representative images of lacunae taken by confocal microscopy. Data presented as mean ± SEM, tested by three‐way ANOVA, significance accepted at *p *< 0.05. Sample size for C‐G: male Control_6‐12_=5, male iGHRKO_6‐12_=5, female Control_6‐12_=5, female iGHRKO_6‐12_=5, male Control_6‐22_=4, male iGHRKO_6‐22_=5, female Control_6‐22_=13, female iGHRKO_6‐22_=9, male Control_6‐30_=5, male iGHRKO_6‐30_=3, female Control_6‐30_=5, female iGHRKO_6‐30_=5

Cortical bone morphology revealed significant decreases in relative cortical bone area (RCA=B. Ar/T. Ar) that were exacerbated with induction of somatopause in male iGHRKO mice at all ages, and at 30 months in iGHRKO_6‐30_ females (Figure 4A). Reductions in RCA with age were accompanied by significant increases in cortical bone porosity (by mCT) that was independent of somatopause (Figure 4B). Reductions in RCA and increased porosity can potentially affect bone matrix organization, osteocyte density and connectivity. Confocal microscopy studies of the osteocyte lacunar‐canalicular network (LCN) in diaphyseal cortical bone revealed that the number of lacunae (Lac.N, #/mm^3^) did not change significantly with age in either male or female control mice (Figure 4C). Induction of somatopause resulted in substantial changes in lacunar number that differed with sex. In female iGHRKO mice, lacunar number (Lac.N) did not change up to 22 months of age, and was not different from aging controls, but Lac.N declined markedly (~22%) by 30 months of age. In male iGHRKO mice, Lac.N declined ~13% between 12 and 22 months, and further declined by ~15% by 30 months of age. Volume of lacunae (Lac.V) increased in iGHRKO as compared to control mice, but reached significance at 12 months only. In female mice, Lac.V varied and showed no difference among mice of any group (Figure 4D).

Canalicular density (Cn.N, 1/µm^3^) in the compact bone was measured as an overall index of osteocyte lacunar connectivity, and canaliculi per lacunae (Cn/Lac, #/Lac) measured to assess changes at the unit cell level (Figure 4E,F). In control aged female mice, neither Cn.N nor Cn/Lac changed up to 22 months of age; however, there was ~22% loss of overall connectivity and ~21% loss of Cn.N by 30 months of age. Female iGHRKO bones showed similar aging trends. In contrast, male control mice exhibited increased Cn.N and Cn/Lac by ~70% from 12 to 22 months, with no change from 22 to 30 months. Canalicular connectivity in aging male iGHRKO mice was highly variable and thus no clear changes could be established.

Finally, we measured area devoid of lacunae and canaliculi from Z‐stack images through 30 µm depth of the cortex with images collapsed into a single merged image using a maximum intensity projection. The area of cortical bone without osteocytes was increased nearly three‐fold in aged female mice (*p *< 0.03 vs. 12 or 24 months old) and by ~1.5‐fold in aged male mice (that did not reach significance). Induction of somatopause did not affect the accumulation of these “acellular” foci of compact bone (Figure 4G).

### 2.3 Induction of somatopause in adult mice did not alter bone matrix composition

**FIGURE 5:**
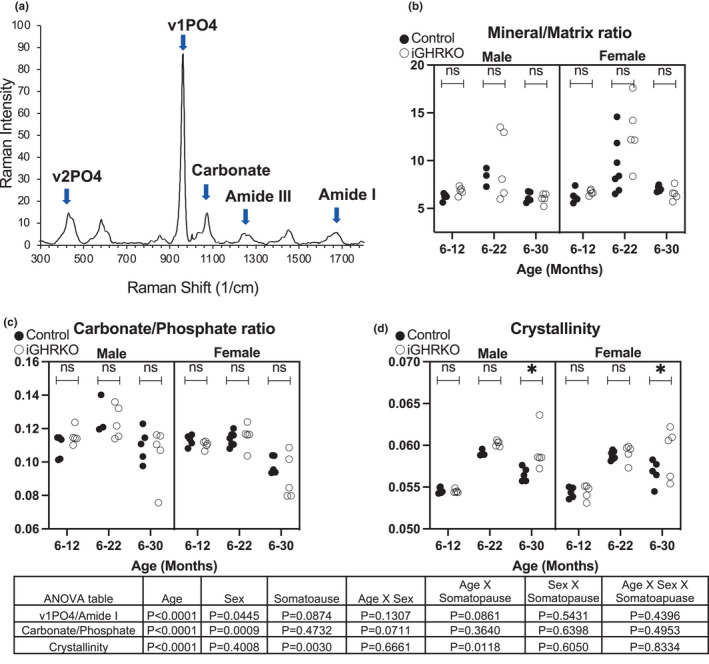
Bone matrix composition by surface‐enhanced Raman spectroscopy. (a) A typical Raman spectrum shows characteristics related to the internal modes of the phosphate (PO_4_^−3^) in the matrix. (b) Mineral/matrix ratio (n1PO_4_ [950–970] /amide I [1660–1690]). (c) Carbonate/phosphate ratio (CO3^−2^ [1050–1070]/ n1PO_4_ [950–970]) (d) mineral crystallinity. Data presented as mean ± SEM, tested by three‐way ANOVA, significance accepted at *p*<0.05. N=5‐7/genotype/sex

Raman microspectroscopy was used to examine how the compositional properties of cortical bone tissue differed with aging and with induction of somatopause among control and iGHRKO mice, respectively. A typical Raman spectrum for mouse femur diaphyseal cortical bone is shown in Figure 5A. Raman spectra of cortical bone were analyzed to compare the means of the three compositional parameters mineral‐to‐matrix ratio, carbonate‐to‐phosphate ratio, and crystallinity across groups. Mineral/matrix ratio varied largely specifically in 22‐month‐old mice (Figure 5B). As expected, mineral/matrix ratio increased with age and peaked at 22 months of age in both male and female mice regardless of somatopause. Mineral/matrix ratio was decreased by more than 25% in 30‐ versus 22‐month‐old mice and this change was independent of somatopause. Carbonate/phosphate ratio, which indicates carbonate substitution for phosphate group in the bone mineral with crystal maturation (Tarnowski et al., 2002), increased in all male bones from 12 to 22 months of age and then dropped significantly (~15%) by 30 months in both control and iGHRKO mice. Carbonate/phosphate ratio was similar in female mice up to 22 months of age, and then declined by almost 15% by 30 months of age in both control and iGHRKO mice (Figure 5C). Crystallinity increased in both sexes between 12 and 22 months of age and this was somatopause independent. Both aged male and female control mice showed decreased crystallinity at 30 months of age. In contrast, crystallinity in iGHRKO mice (both sexes) was not reduced at 30 months of age (Figure 5D). Overall, induction of somatopause in adult mice had no significant effects on matrix mineral content. Mineral crystallinity, which reflects crystal size and perfection, was significantly reduced in controls but not in mice with induced somatopause at 30 months of age.

### 2.4 Induction of somatopause impairs the bone marrow microenvironment

**FIGURE 6:**
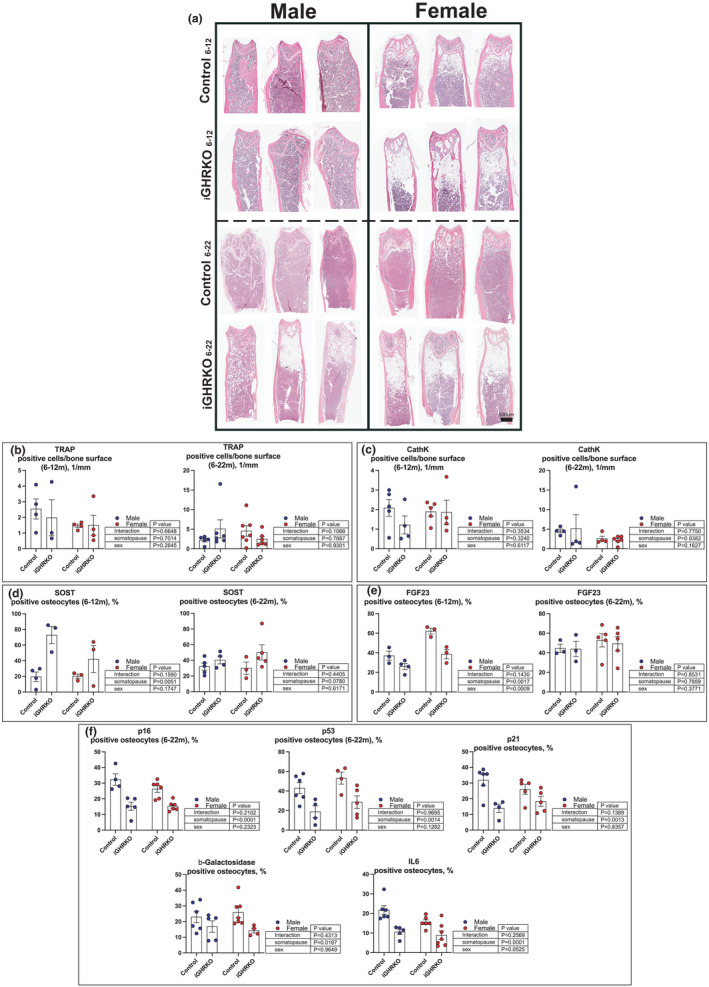
Somatopause‐induced bone marrow adiposity: Bones were decalcified, embedded in paraffin, and sectioned for histology and IHC. (a) Bone marrow adiposity was studied in iGHRKO mice with age‐induced somatopause by histology. In accordance with the findings in constitutive GH resistance, both male and female iGHRKO mice showed increased bone marrow adiposity. (b) TRAP‐positive cells on femur subchondral bone surface. (c) Cathepstin K‐positive cells on femur subchondral bone surface. (d) Sclerostin, (e) FGF23, (f) p16, p53, p21, β‐galactosidase, and IL6‐positive cortical osteocytes. Data presented as mean ± SEM, tested by two‐way ANOVA, significance accepted at *p *< 0.05. N=5‐7/genotype/sex

To further investigate the bone microenvironment, we used histology and immunohistochemistry (IHC). H&E stain revealed increased bone marrow adipocytes in both male and female iGHRKO mice, detected at 12 and 22 months of age (Figure 6A). Increased bone marrow fat is expected to accelerate the inflammatory milieu, drive osteoclastogenesis, and induce bone resorption. To detect osteoclast activity, we stained bone sections with tartrate acid phosphatase (TRAP), that is highly expressed in osteoclasts (Figure 6B, Figure S5) as well as with cathepsin K, an osteoclast marker (Figure 6C, Figure S5). We did not find significant differences in the number of TRAP or cathepsin K‐positive cells on the trabecular surface of subchondral bone at the distal femur in both sexes. Bone remodeling is orchestrated by osteocytes that signal to osteoblasts and osteoclasts on bone surfaces. Osteocytes control bone remodeling via expression of sclerostin (SOST), a negative regulator of bone formation, and phosphate metabolism via expression of fibroblast growth factor‐23 (FGF23). Interestingly, we found significantly increased sclerostin and decreases in (FGF23) levels at 12‐month‐old male iGHRKO mice (Figure 6D,E). Female iGHRKO mice showed similar trends that did not reach significance.

Finally, accumulation of senescent cells has emerged as one of the characterizations of the aging fat pads (Tchkonia et al., 2010). A previous report using the constitutive GHRKO mice has shown reduced senescence in white adipose tissue despite increased fat mass (Stout et al., 2014). Since we detected increased bone marrow adiposity in the iGHRKO mice, and based on the roles played by GH in adipose tissues and the bone marrow (Menagh et al., 2010) we were interested whether the iGHRKO mice were protected from senescence despite increased marrow adiposity. We determined the levels of p16, p21, p53, β–galactosidase, and the inflammatory marker, interleukin‐6 (IL6), in cortical osteocytes (Figure 6F, Figure S5). Surprisingly, we found decreases in those senescence markers in iGHRKO mice at 22 months of age, despite increases in marrow adiposity, indicating that reduced GH action in the bone and the bone marrow may have protective roles against senescence.

## 3 DISCUSSION

Using a unique mouse model of induced somatopause in skeletally mature mice, we show that declines in GH/IGF‐1 actions, specifically during chronological aging, inhibit radial bone expansion, an important determinant of bone strength, in both sexes. Induction of somatopause minimally affected the osteocyte LCN or the bone matrix mineralization, but was associated with increases in bone marrow adiposity. Despite significant decreases in bone volume at both the cortical and the trabecular bone compartments, we did not detect increased resorption markers by histology at 12 or 24 months of age. However, the levels of sclerostin, an endogenous inhibitor of bone formation, significantly elevated in mice with induced somatopause. Remarkably, however, as seen in fat pads of the constitutive GHRKO mice (Stout et al., 2014), we found that mice with induced somatopause exhibited reduced osteocyte senescence.

Unlike humans who show gradual decrease in both GH and IGF‐1 levels during aging (Garcia et al., 2019), mice show reduced GH secretion with age, but IGF‐1 levels remain unchanged from 12 months onward (Yuan et al., 2009). Thus, chronological aging in mice does not resemble human aging, which is accompanied by somatopause. To study how somatopause drives bone fragility in conjunction with aging, we induced somatopause globally after peak bone acquisition in mice (iGHRKO). This model is based on a ubiquitously expressed Cre that once activated by tamoxifen (at 6 months of age), ablates the *GHR* gene in all tissues including the bone and the bone marrow (Figure S6), consequently leading to reductions in IGF‐1 in serum and peripheral tissues (Junnila et al., 2016; Silvana et al., 2021). Notably, recombination efficiency did not achieve 100% in all tissues (>90% in liver, 50%–70% in all other tissues), nevertheless, overall reductions in the GH/IGF‐1 signaling was sufficient to cause deleterious effects on bone. Importantly, compared with the constitutive GHRKO mice that show marked reductions in body weight, three‐ to five‐fold increases in fat mass (Berryman et al., 2004,2010; Stout et al., 2014; Wang et al., 2006) and extreme insulin sensitivity (Gesing et al., 2017), the skeletally mature mice, induced with somatopause at 6 months, showed similar body weight, with initial ~15% increases in fat mass and ~10% decreases in lean mass (in both male and female iGHRKO mice) between 6 and 22 months of age that was normalized thereafter (Silvana et al., 2021). Thus, the iGHRKO is a good model to study the interactions between somatopause and chronological aging.

Consistent with the literature, aging in control male mice was associated with progressive endocortical resorption and bone formation on the periosteal surface, leading to cortical thinning and expansion of the marrow cavity (Boskey & Coleman, 2010). Induced somatopause, however, caused uncoupling of these two processes in male iGHRKO mice. This was evidenced by similar T. Ar in iGHRKO_6‐22_ and iGHRKO_6‐30_ males (no increase in periosteal expansion), and significantly reduced Ct. Th (endocortical resorption). In control females, we observed increased endocortical resorption and periosteal formation with age, but significant cortical thinning was seen only between 22 and 30 months of age. Somatopause in females, resulted in the inhibition of endocortical resorption (M. Ar) and periosteal expansion (T. Ar). The fact that the effects of somatopause on bones in female iGHRKO mice manifested later in life may relate to their increase in lifespan and decreased senescence in their bone tissue. Alternatively, we speculate that it may relate to their sex steroid state. It is well established that mice aged around 9–12 months typically experience irregular estrous cycle (estropause) that shifts into anestrous state (around 20–24 months) (Koebele & Bimonte‐Nelson, 2016). It is plausible that the transition from estropause to anestrous lasts longer in iGHRKO females or that they experience less reproductive senescence that indirectly affects their estrogen levels that play protective roles in the bone tissue. However, validation of these speculations will require more comprehensive analyses.

Cortical morphology in the iGHRKO mice resembled as that seen in mice with constitutive GHRKO in mature osteoblasts and osteocytes, using the dentin matrix protein‐1 (DMP‐1)‐specific Cre (DMP‐GHRKO), which also showed slender bones during development and maturation (Liu et al., 2016). Similarly, mouse models with constitutive reductions in serum IGF‐1, such as the liver IGF‐1‐deficient (LID) mice (Yakar et al., 2009), the liver‐specific GHRKO (Liu et al., 2018), and the acid‐labile subunit (ALSKO) null mice (Courtland et al., 2010), all showed slender bones with inhibited periosteal expansion. All together, these mouse lines suggest that the GH/IGF‐1 axis is essential for regulating both linear and radial bone expansion, not only during growth but also during aging. Finally, as seen in other studies (Ding & Hvid, 2000; Majumdar et al., 1997), changes in trabecular bone included decreases in trabecular number, increased trabecular spacing, and unaffected trabecular thickness with progression of age, independent of induced somatopause.

Besides bone geometry, that determines mechanical properties at the macrolevel, tissue‐level morphology, including microporosity and the osteocyte lacunar‐canalicular network (LCN), play significant roles in bone tissue quality or local material properties (Hazenberg et al., 2007; Ma et al., 2008; Schneider et al., 2013; Voide et al., 2011). The LCN is a continuous interstitial fluid pathway through which osteocytes obtain nutrients and dispose of wastes. Transport of intercellular signaling molecules through the LCN is also an important means of communication among osteocytes (Genetos et al., 2007; Kennedy et al., 2012; Schaffler et al., 2014). Importantly, changes in lacunar density along the cortex determine the mechanical behavior of the bone by either dissipating energy to slow down microcrack propagation (O'Brien et al., 2003), or by acting as local stress concentrators that cause crack initiation (Bonivtch et al., 2007; Nicolella et al., 2006). Interestingly, in males, induced somatopause caused progressive decline in lacunar density along the cortex, while in females a marked reduction in lacunar density was seen only during advanced age (between 22 and 30 months). Reductions in osteocyte density with age and somatopause can possibly contribute to decreased bone resistance to fracture and impaired tissue repair (Burger et al., 2003; Ma et al., 2008; Mullender et al., 2005; O'Brien et al., 2004). Changes in osteocyte shape also contributes to bone mechanical behavior (Nicolella et al., 2006). In humans, it was shown that osteocytes became more spherical with age (Carter et al., 2013). However, studies in mice show conflicting results that are bone specific (Heveran et al., 2018). We found that osteocyte shape was highly variable ranging from a clear ellipsoid to spherical shape. The variations in shape and alignment of osteocyte lacunae possibly reflect physiological adaptation to micromechanical stressors in bone throughout life. Another determinant that controls bone mechanical behavior at the microlevel is osteocyte connectivity, namely the canaliculi network/density. Presumably, loss of canaliculi per lacuna would reduce the mechanosensitivity of the osteocytes (Bonivtch et al., 2007). In our model, the number of canaliculi per lacuna varied in both sexes of control and iGHRKO mice. Indeed, somatopause in females caused ~25% loss of overall connectivity by 30 months of age. However, variation in canalicular density was high in males and even increased with early aging (12–22 months). Greater variability in canalicular density in male mice has been shown previously (Tiede‐Lewis et al., 2017) and may reflect their rapid periosteal expansion early in life (Callewaert et al., 2010). Finally, lacunar and canalicular measures do not reflect a complete picture of how osteocytes and their connectivity are altered with age. We identified regions in the aged bones that were devoid of canaliculi and lacunae, pointing to focal regional losses of osteocytes during aging. These acellular foci were increased with age in both sexes, but were independent of induced somatopause.

As indicated above, decreased osteocyte density or loss of connectivity (i.e., loss of canaliculi) can result in bone tissue compositional changes. As such, accumulation of mineral in lacunae (micropetrosis) with aging can significantly compromise bone strength, leading to decreased energy absorbing and dissipating capacities of the bone tissue (Hemmatian et al., 2017; Klein‐Nulend, 2008). However, Raman microspectroscopy of cortical bone indicated no significant differences in mineral to matrix ratio between genotypes or ages. Mineral crystallinity increased with age in both sexes, independent of somatopause, but was reduced dramatically at 30 months of age, significantly more in controls than in iGHRKO mice. There are many intrinsic and extrinsic factors that influence the size and composition of the mineral crystals in bone including the organization of collagen, the distribution of matrix proteins, bone turnover, among others (Adele, 2003), however, these were not investigated in the current study. Overall, in accordance with our findings by mCT, which showed no differences in tissue mineral density between the groups, the Raman spectra did not reveal significant differences in mineralization between control and iGHRKO mice in both sexes.

Increased body (Bonkowski et al., 2006) and bone marrow adiposity (Menagh et al., 2010) in both sexes of the constitutive GHRKO mice is well described. Importantly, here we describe significant increases in bone marrow adiposity in the iGHRKO mice, where activity of the GH/IGF‐1 axis was ablated after skeletal maturity. These data suggest that the GH/IGF‐1 axis regulates mesenchymal stem cell lineage commitment to osteogenic or adipogenic fate, not only during growth but also during aging. Marrow adiposity was found in both sexes but was more profound in female iGHRKO mice. Although yet to be established, a link between bone marrow adiposity and expression of sclerostin in osteocytes was described *in vivo* and *in vitro* (Fairfield et al., 2017). Transgenic mice overexpressing sclerostin show increased body adiposity (Kim et al., 2017), and recombinant sclerostin stimulated differentiation of 3T3‐L1 pre‐adipocytes, in a dose‐dependent manner (Ukita et al., 2016). We found increases in sclerostin levels in osteocytes by IHC in both male and female iGHRKO mice that may partially explain the inhibited periosteal bone formation. Additionally, adipocytes can impact the bone microenvironment via secretion of adipokines that inhibit osteogenesis (reviewed in Muruganandan et al., (2018)).

Finally, we estimated the level of osteocyte senescence *in situ* by IHC of several markers. The overall percentage of senescent cells in aged tissues is usually <20% (Yang & Sen, 2018), and increases with age (Biran et al., 2017; Herbig et al., 2006; Mishima et al., 1999). Despite their small number, senescent cells amplify their damage to the surrounding tissues via paracrine secretion of inflammatory cytokines. We note that detecting and assessing cellular senescence *in vivo* with high levels of confidence remains a challenge (Gonzalez‐Gualda et al., 2021). The heterogeneity of senescent cells and the intrinsic complexity of a living organism especially during aging hinder the precise evaluation of senescence *in vivo*. Nevertheless, we report reductions in β‐galactosidase, p16, p21, p53, and IL6 in iGHRKO mice as compared to controls. These results are in line with findings in fat tissue of the long‐lived constitutive GHRKO mice (Stout et al., 2014), and may contribute to the increased lifespan and the delayed bone phenotype in female iGHRKO mice. Cautiously however, the interactions between the GH/IGF‐1 axis and senescence during aging are complicated and may be tissue‐ and sex specific. We have recently reported a mouse model with adult‐onset inducible GH deficiency (AOiGHD) that was associated with increased lifespan in female, but not male mice (Poudel et al., 2021). In this model, both male and female AOiGHD mice showed increased chondrocyte senescence in the articular cartilage that was associated with severe osteoarthritis. We believe that the lifelong and complex interactions between tissues, in models with constitutive GH inactivation, improve overall metabolic homeostasis and by inference health span. In contrast, significant inhibitions of GH/IGF‐1 actions during aging *per se* play detrimental roles in specific tissues.

In summary, our results challenge the accepted notion that reductions in GH/IGF‐1 extend lifespan and by inference, health span. Data presented herein strongly indicate dissociation between lifespan and health span, specifically in the skeleton. Induced somatopause significantly increased lifespan in female iGHRKO mice (Silvana et al., 2021), but impaired bone morphology, and significantly increased the bone marrow adiposity. In contrast, male iGHRKO mice, that do not show increased lifespan, exhibit enhanced health span evidenced by increased insulin sensitivity and decreased overall oxidative stress (Silvana et al., 2021), but their bone morphology was deteriorated earlier than female iGHRKO mice. Overall, we show that somatopause, induced specifically during aging, exacerbates bone morphology and significantly alters the bone marrow microenvironment, implying that aged subjects with significant reductions in GH/IGF‐1 are at higher risk for bone pathologies.

## 4 METHODS

### 4.1 Animals

#### 4.1.1 Ethics statement

All procedures involving mice were reviewed and approved by the Institutional Animal Care and Use Committee of The Ohio University, Southern Illinois University School of Medicine, and The NYU School of Medicine.

#### 4.1.2 Mice

Generation of the iGHRKO mice was previously described (Junnila et al., 2016). All mice were in the C57BL/6J (B6) genetic background. Mice were housed at 22°C under a 14‐hour light, 10‐hour dark cycle, three to four mice per cage, and ad libitum access to water and standard laboratory chow (ProLabRMH3000). To induce Ghr gene ablation, 6‐month‐old mice received 100‐µl ip injections of 0.32 mg of Tam/g of body weight dissolved in peanut oil (25) once per day over five consecutive days, for a total of 5 mg of tamoxifen (26). Controls received identical injections of peanut oil. Mice were dissected at three time points; 12, 22, and at their end of life, ~30 months of age. Tissue dissections took place in the morning after a 12‐hour overnight fast followed by exposure to CO_2_ until they became unconscious. Mice were killed by cervical dislocation. Animals that were sacrificed at 12 and 22 months of age were selected randomly from their groups and did not show signs of morbidity/pathology. Mice that were reported in the X_6‐30_ groups were euthanized and dissected when found severely moribund, or shortly after death. Mice were only used for analysis if death occurred less than 24 h before collection. Constitutive (congenital) GHRKO was previously described (Fang et al., 2020).

### 4.2 Micro‐computed tomography

Micro‐CT was done in accordance with the American Society for Bone and Mineral Research (ASBMR) guidelines (Bouxsein et al., 2010). Hind limbs were dissected and fixed in 10% formalin, cleaned to remove skin and extra tissues, transferred to 70% ethanol, and stored at 4°C until the time of analysis. Limbs were scanned using a high‐resolution SkyScan micro‐CT system (SkyScan 1172, Kontich, Belgium) containing 10‐M digital detector set at a 10W energy level (100kV and 100 μA), with a 0.5‐mm aluminum filter with a 9.7‐μm image voxel size. Femur cortical bone was analyzed in a 2.0‐mm femur mid‐diaphyseal region directly below the third trochanter. Measurements included total cross‐sectional area inside the periosteal envelope (T. Ar, mm^2^), cortical bone area (B. Ar, mm^2^), cortical cross‐sectional thickness (Ct. Th, mm), and bone mineral density (BMD, g/cc). We used 3D analysis of the 2‐mm mid‐diaphyseal region (that was analyzed for cortical parameters) to calculate total cortical bone porosity (%). Following “despeckle and shrink‐wrap” steps in 3D to remove noise, we wrap the region of interest tightly within the boundaries of the object, isolated the marrow cavity, and calculated all the closed and open pores included in the cortical envelope. Trabecular bone parameters were taken at the femur distal metaphysis in a 2.5‐mm region below the growth plate and included bone volume fraction (bone volume/tissue volume, (BV/TV %), trabecular thickness (Tb. Th, mm), trabecular number (Tb.N, 1/mm), and bone mineral density (BMD, mg/cc). Data reconstruction was done using NRecon software (version 1.7.3.0; Bruker micro‐CT, Kontich, Belgium), data analysis was done using CTAn software (version 1.17.7.2+; Bruker micro‐CT, Kontich, Belgium), and 3D images were done using CT Vox software (version 3.3.0 r1403; Bruker micro‐CT, Kontich, Belgium).

### 4.3 Raman microspectroscopy

Tissue processing: Femurs, fixed in 10% formalin, were sectioned (500 µm thickness) at the mid‐diaphysis, immediately distal to the third trochanter, using a low‐speed diamond‐coated wafering saw (Buehler). Specimens were dehydrated and embedded undecalcified in poly‐methyl methacrylate (PMMA). Two hundred‐micron sections were adhered to plastic slides using Eukitt's mounting medium and used for *Raman Microspectroscopy*.

*Raman Microspectroscopy* : We used the Thermo‐Fisher DXR2 confocal Raman microscope, with 785‐nm wavelength excitation at 50X/0.75 BD objective, a 50‐µm entrance slit provided a spectral resolution of 50–3300 cm^−1^ and a 0.96‐µm diameter spot size. This spot size was small enough to avoid including osteocyte lacunae visible on the bone surface in the measurements, allowing us to capture Raman spectra in interlacunar bone areas that contained only matrix and canaliculi.

Spectra were collected from middle of the cortex of the anterior quadrants of the bone, as in our previous studies (Kaya et al., 2017). Raman spectra (n = 5–10 per section, dictated by cortical width) were captured from the anterior quadrant of each femur cross‐section, examined, and averaged per bone. Each spectrum was acquired at the mid‐cortex width location at 20‐µm distance from each other. Data acquisition and analysis were performed using OMNIC spectroscopy software. Spectra were normalized with respect to strongest phosphate band area and PMMA background was subtracted using the system software. The following parameters were determined as described by Morris et al (Tarnowski et al., 2002): (1) Mineral/Matrix ratio, which measures the degree of mineralization of the tissue; (2) Carbonate/Phosphate ratio, which is an indication of carbonate substitution for phosphate in bone crystal lattice; and (3) Mineral Crystallinity, an indicator of mineral crystal size and/or lattice perfection. Three band areas were determined: phosphate band (960 cm^−1^), carbonate band (1070 cm^−1^), and Amide I band (1620–1700 cm^−1^). Mineral/matrix ratios were obtained by dividing the integrated band areas of phosphate to amide I. Carbonate/phosphate ratios were calculated by dividing carbonate band areas by phosphate band areas. Mineral crystallinity information was determined by taking the inverse of full width at half maximum (FWHM) for the phosphate band (crystallinity=1/FWHM).

### 4.4 Characterization of the lacunar canalicular network

Femurs, fixed in 10% formalin, were sectioned (500‐μm thickness) at the mid‐diaphysis, immediately distal to the third trochanter, using a low‐speed diamond‐coated wafering saw (Buehler). Specimens were dehydrated and embedded undecalcified in poly‐methyl methacrylate (PMMA) mixed with Pylaklor Pink S351 (Pylam Products) fluorescent dye. Two hundred‐micron sections were adhered to plastic slides using Eukitt's mounting medium and used for imaging. Confocal laser scanning microscopy image stacks were obtained with a Zeiss LSM 800 with 561 nm excitation and a 40× oil immersion objective. Images stacks were taken to capture the entire cortical thickness (~74–306 μm), and were 200 μm radially and 30 μm thick (0.088 μm per pixel, 0.480 μm between slices). Two images each from the anterior and posterior cortex of each animal were analyzed using an automatic custom software written in MATLAB (v2021a, MathWorks) using methods similar to previous studies on the morphology of the LCN (Heveran et al., 2018; Wittig et al., 2019). Briefly, lacunae were segmented using local thresholding, opened to remove canaliculi, volume filtered to remove small noise and large vessels, and cells touching the edges were removed. Lacunar density was then calculated as the number of lacunae in a given volume of bone, and lacunar volume and surface area were determined using methods introduced by Heveran and colleagues (Heveran et al., 2018). Then, the number of canaliculi on each lacuna was determined by counting the number of overlapping blobs between the original thresholded image and the lacuna with one dilation. Canalicular density was then calculated as the average number of canaliculi over the surface area of a lacuna. Areas devoid of lacunae and canaliculi were quantified from stacks of images that were collapsed using a maximum intensity projection in ImageJ 1.53c using Fiji (Schindelin et al., 2012; Schneider et al., 2012). Gaps due to resting lines were manually excluded, and then, a global threshold was applied to determine the percentage of sample area that included gaps or holes.

### 4.5 Histology

Femurs were fixed in 10% formalin, decalcified in 10% EDTA for 4 weeks, and dehydrated using graded alcohol series and xylene, and processed for paraffin embedding and sectioning. Sections (7 µm) were stained with H&E and TRAP (Sigma, 387A‐1KT). For immunostaining, sections were treated with 3% hydrogen peroxide to block the endogenous peroxidase. The antigen retrieval was done by antigen unmasking solution following the instruction provided by the manufacturer (H‐3300; Vector Laboratories, CA, USA). The sections were incubated with 5% bovine serum albumin for 30 min at room temperature, then with primary antibodies to Cathepsin K (1:200, #ab19027, abcam), p16 (1:100; #PA30670, Invitrogen), p53 (1:100; # ab31333, Abcam), FGF23 (1:100; # MAB26291, R & D systems), SOST (1:12; # AF1589, R & D systems), overnight at 4 °C. Incubation with HRP‐conjugated secondary antibody (Cat. No. PK4001, Vector Laboratories, CA, USA) was done for 1 hour at RT. Immunoreactivity was detected using the horseradish peroxidase‐3,3′‐diaminobenzidine system (SK‐4100 vector laboratories, USA) followed by counterstaining with hematoxylin (Sigma). The images were acquired by Aperio CS2 Scanner (Leica Biosystems) and analyzed by Fiji ImageJ (version 1.51r; NIH).

### 4.6 Statistical analyses

Data presented as mean ± SEM, and significance accepted at *p*<0.05. For the comparison between groups, data were analyzed by three‐way analysis of variance (ANOVA) (GraphPad Prism version 9.0) with age, sex, and induction of somatopause as independent variables. In cases where only one age was analyzed (IHC), two‐way ANOVA was applied, and this was indicated in the figure legends. Statistical tests for LCS parameters were performed in RStudio (version 1.3.959, RStudio Team 2020) running R (version 4.0.2, R Core Team 2020) using the *rstatix* package [R package version 0.6.0. https://CRAN.R‐project.org/package=rstatix]. For measurements taken repeatedly in a scan (lacunar volume, canalicular density), an average was obtained for each of the four scans, the scan averages were averaged to obtain a value for the animal, which was used in statistical tests. First, an analysis of variance was performed, with age, sex, and induction of somatopause as independent variables. This was followed by post hoc Tukey honestly significant difference tests as needed to determine subgroup differences by age and somatopause.

## CONFLICT OF INTEREST

The authors declare no conflict of interest. All authors have discussed the results and approved the final version of the manuscript. SY is the guarantor of this work and, as such, had full access to all the data in the study and takes responsibility for the integrity of the data and the accuracy of the data analysis.

## AUTHOR CONTRIBUTIONS

Study conception and design: SY, JJK, AB, MBS. Acquisition of data: MD, SDO, GY, SBP, LDL, AB. Analysis and interpretation of data: SY MD, GY, LDL, MBS. Manuscript preparation: SY and MBS.

## STATEMENTS

All authors concur with the submission. The material submitted for publication has not been previously reported and is not under consideration for publication elsewhere.

## Supporting information

Fig S1‐S6

## Data Availability

The datasets generated and analyzed during the current study are available upon request. Our studies do not include the use of custom code or mathematical algorithms. We have included citations for available data in the references section.
