# Supplementary material for: Induction of somatopause in adult mice compromises bone morphology and exacerbates bone loss during aging
Source: Aging Cell. 2021 Nov 23;20(12):e13505. doi: 10.1111/acel.13505 (PMC8672783; doi:10.1111/acel.13505)
Supplement: Supplementary file 1 — Fig S1‐S6 [file ACEL-20-e13505-s001.pdf]

Supplement Figure 1

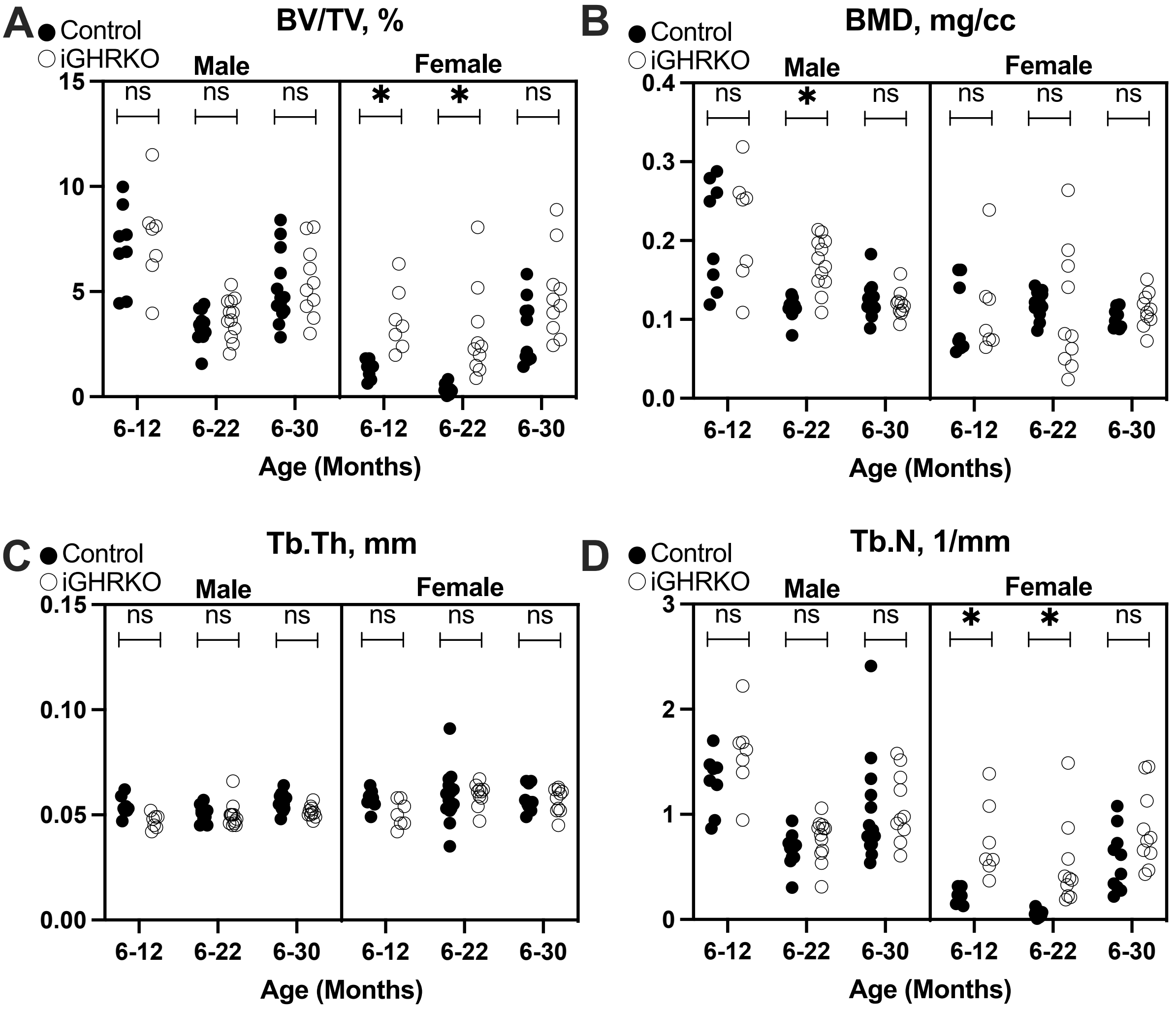

ANOVA table	Age	Sex	Somatoause	Age X Sex	Age X Somatopause	Sex X Somatopause	Age X Sex X Somatoapuase
BV/TV	P<0.0001	P<0.0001	P<0.0001	P<0.0001	P=0.7575	P=0.0018	P=0.7801
BMD	P<0.0001	P<0.0001	P=0.1003	P<0.0001	P=0.5439	P=0.3127	P=0.0463
Tb.Th	P=0.0993	P<0.0001	P=0.0064	P=0.0777	P=0.0795	P=0.4305	P=0.8060
Tb.N	P<0.0001	P<0.0001	P<0.0001	P=0.0001	P=0.3359	P=0.0172	P=0.9120

Supplement Figure 1:
Trabecular bone volume reduces with age, independent of somatopause. (A) Bone volume/toatal volume (BV/TV), (B) bone mineral density, (C) trabecular bone thickness (Tb.Th), and trabecular bone number (Tb.N), were determined at the femur distal metaphysis by mCT of mice at the indicated ages. Data presented as mean+/-SEM, tested by 3-way ANOVA, significance accepted at p<0.05. Sample size: male Control$_{6\text{-}12}$=10, male iGHRKO$_{6\text{-}12}$=7, female Control$_{6\text{-}12}$=10, female iGHRKO$_{6\text{-}12}$=8, male Control$_{6\text{-}22}$=11, male iGHRKO$_{6\text{-}22}$=12, female Control$_{6\text{-}22}$=9, female iGHRKO$_{6\text{-}22}$=9, male Control$_{6\text{-}30}$=17, male iGHRKO$_{6\text{-}30}$=11, female Control$_{6\text{-}30}$=13, female iGHRKO$_{6\text{-}30}$=11.

Supplement Figure 2

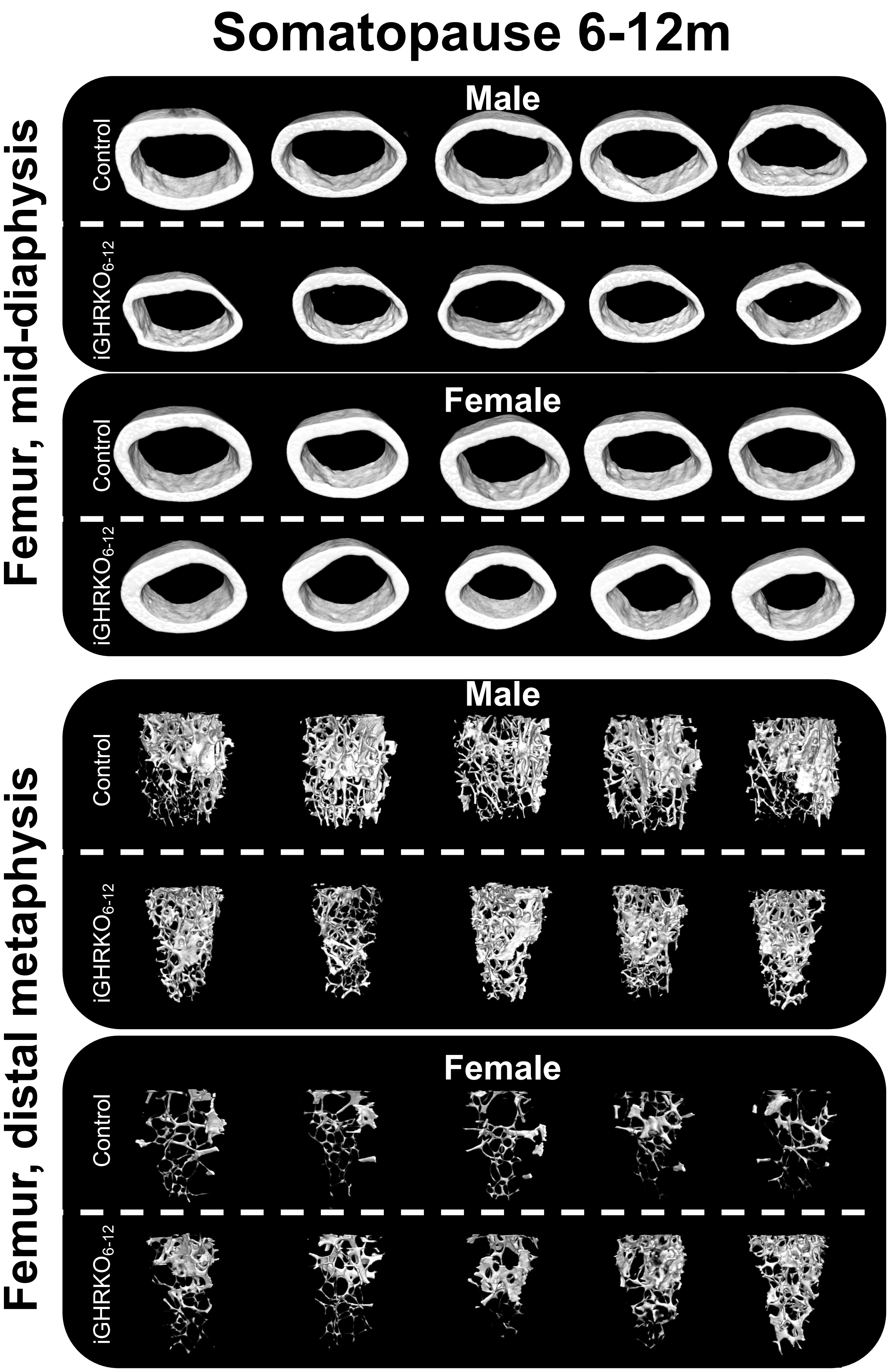

Supplement figure 1: mCT 3D images of the femur mid-diaphysis and the femur distal-metaphysis of control and iGHRKO male and female mice at 12 months of age.

Supplement Figure 3

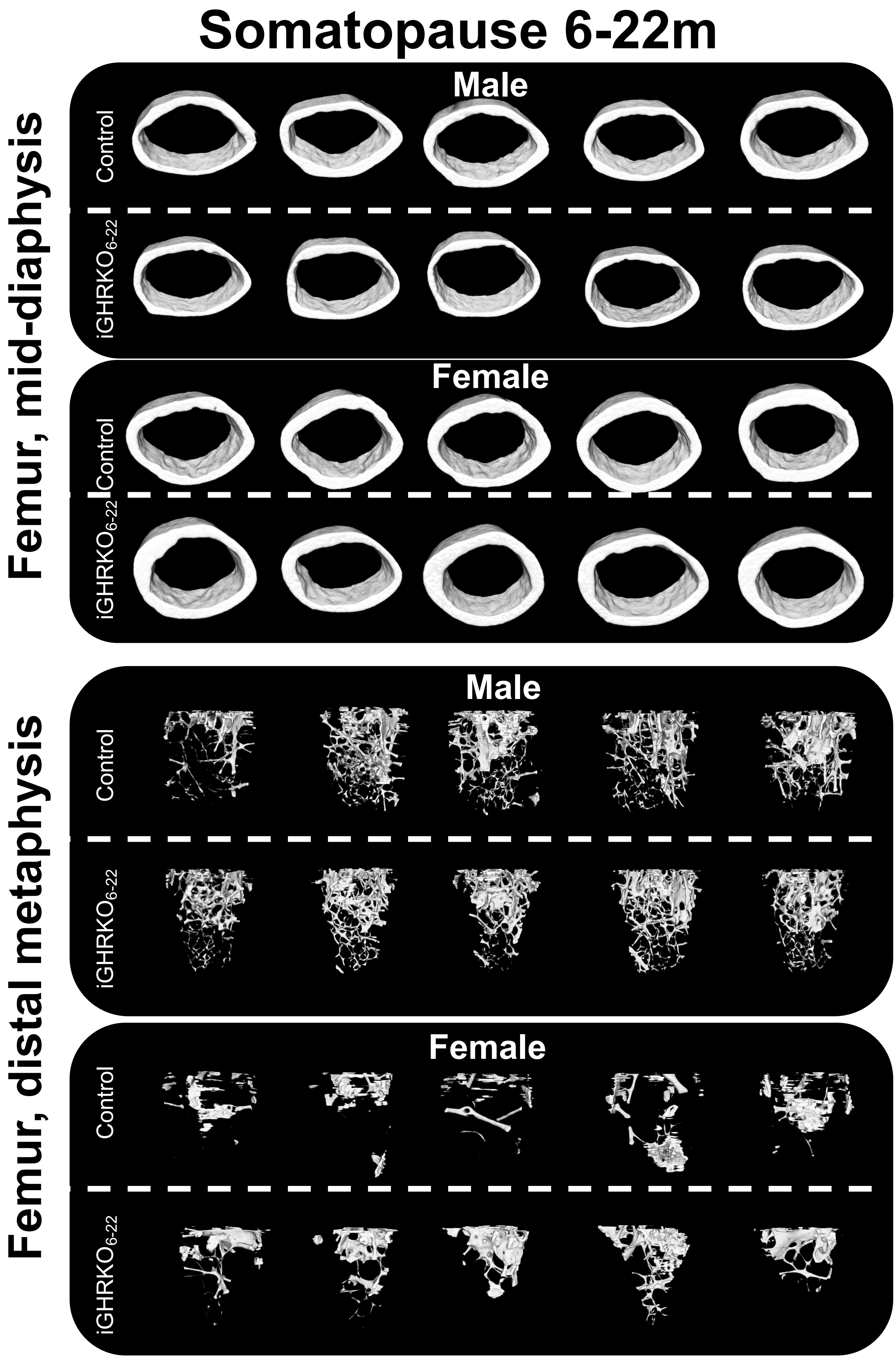

Supplement figure 1: mCT 3D images of the femur mid-diaphysis and the femur distal-metaphysis of control and iGHRKO male and female mice at 22 months of age.

Supplement Figure 4

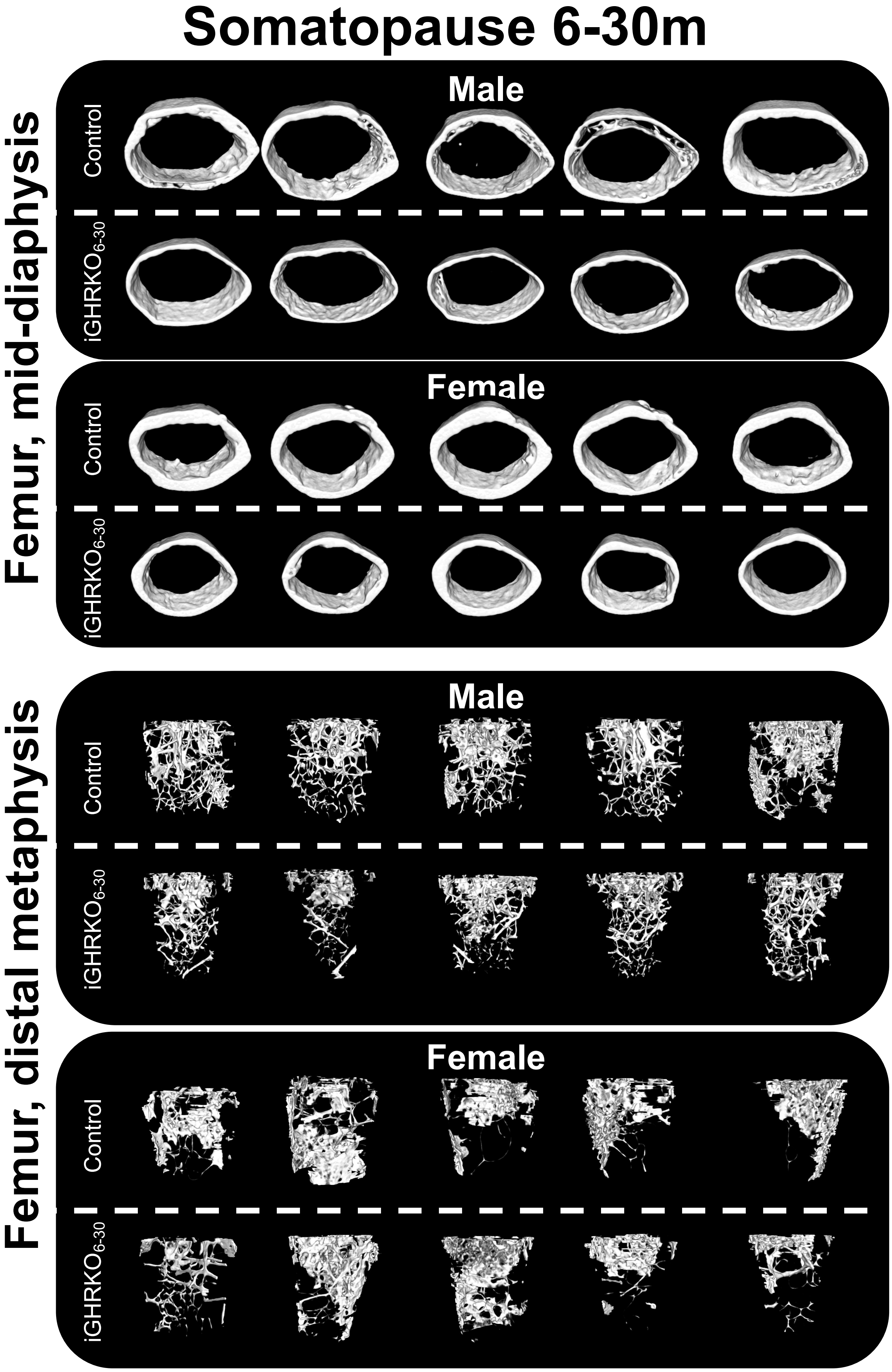

Supplement figure 1: mCT 3D images of the femur mid-diaphysis and the femur distal-metaphysis of control and iGHRKO male and female mice at 30 months of age.

Supplement Figure 5

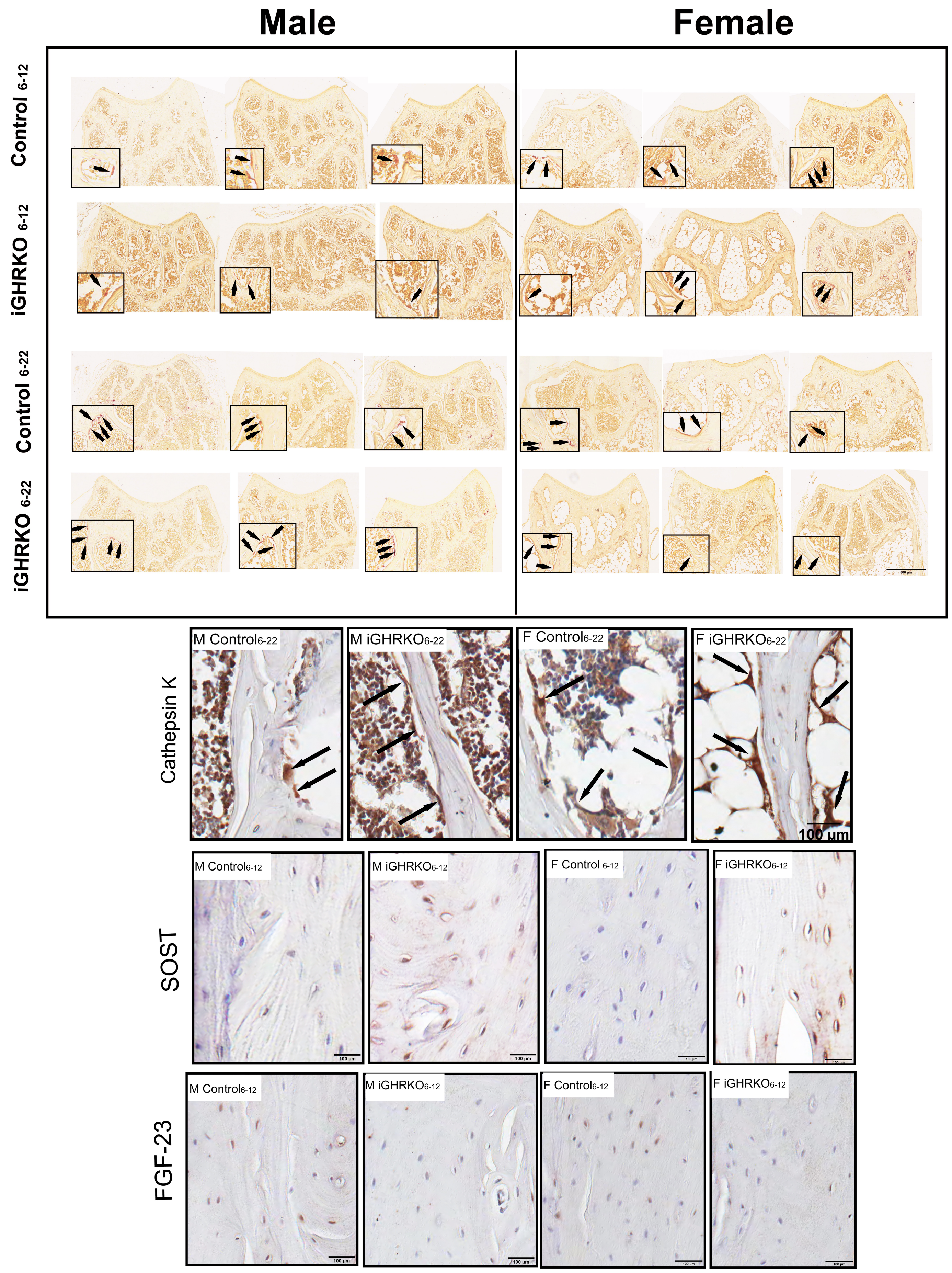

Supplement figure 5 (part 1): Femurs were decalcified, embedded in paraffin and sectioned for 7um, and stained for (A) Tartrate acid phosphatase (TRAP), (B) Cathepsin K (C) sclerostin, and (D) FGF23.

Supplement Figure 5 continue

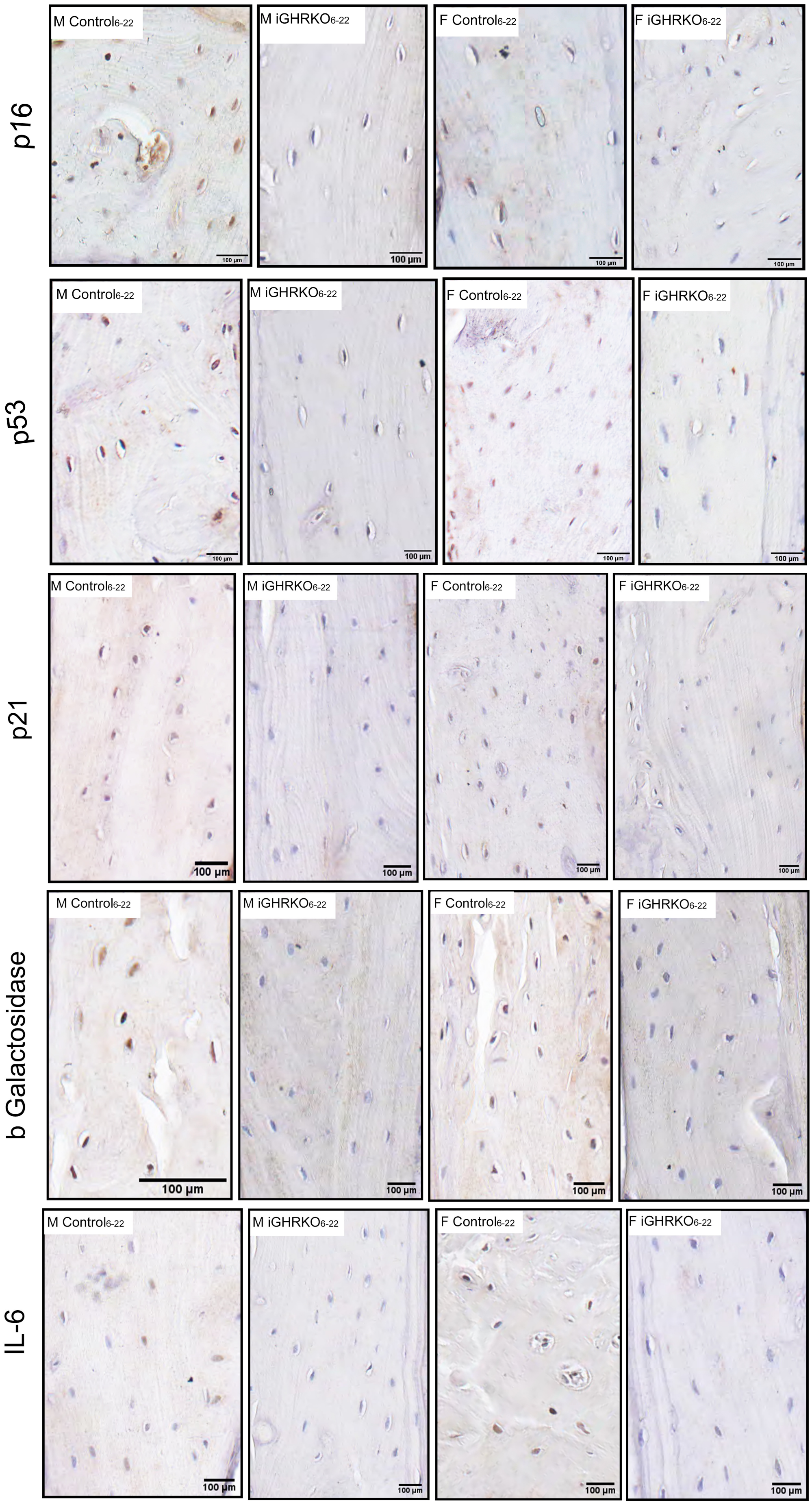

Supplement figure 5 (part 2): Femurs were decalcified, embedded in paraffin and sectioned for 7um, and stained for, (F) p16, p53, p21, b-galactosidase, and IL6 positive cortical osteocytes .

Supplement Figure 6

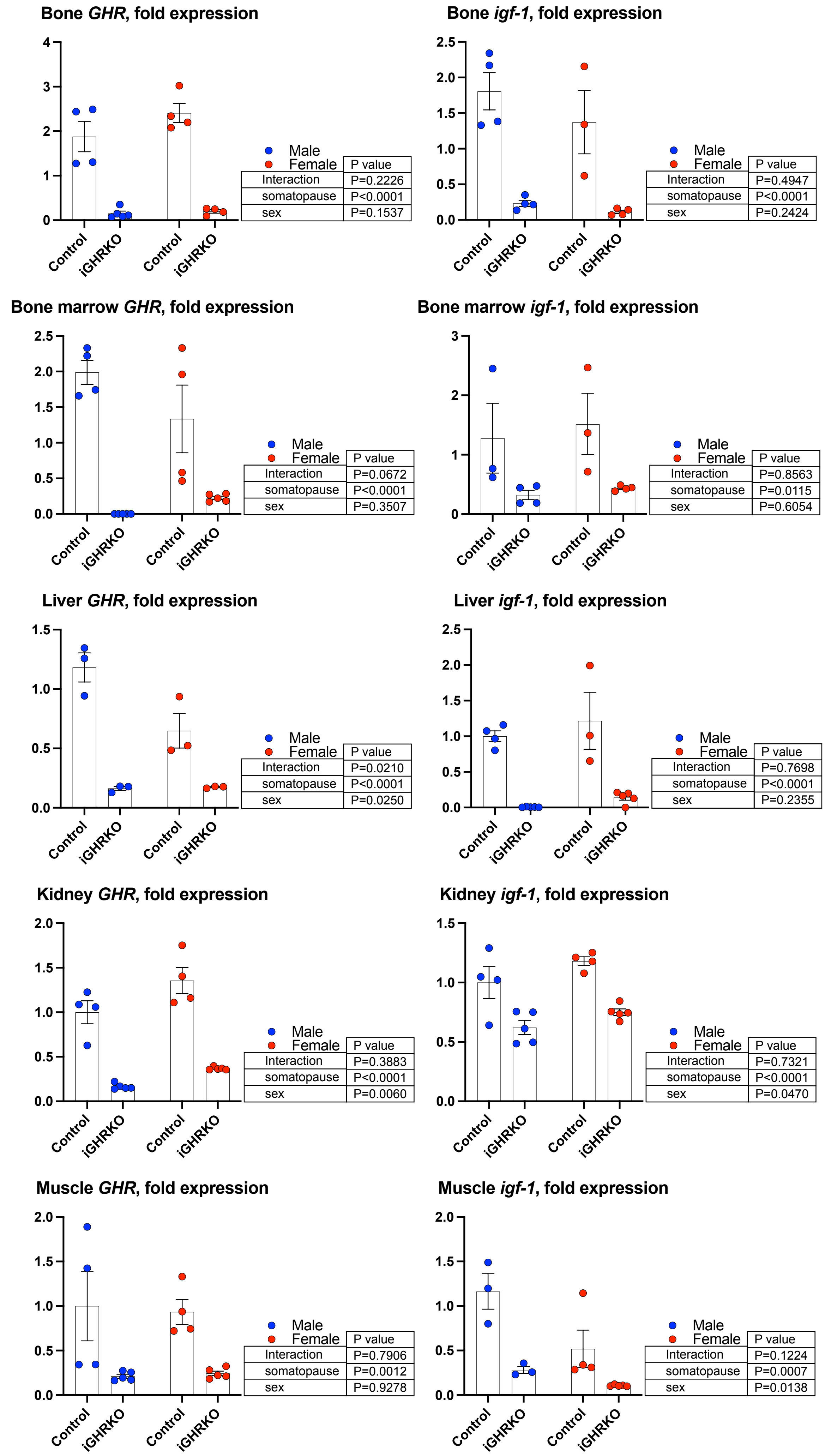

Supplement figure 6:
GHR and IGF-1 expression in key tissues 6 months following induction of somatopause. A separate group of animals was used to demonstrate recombination efficiency. RNA was extracted from bone marrow, femur cortical bone shells (bone), liver, muscle, and kidney using Qiagen RNA isolation kit (cat#174316). One microgram RNA was reversed transcribed to cDNA and subjected to real-time qPCR. The following primers were used: GHR forward: GCCTGGGGACAAGTTCTTCTGGA, GHR reverse: TGCAGCTTGTCGTTGGCTTTCCC, IGF-1 forward: GAGACTGGAGATGTACTGTG, IGF-1 reverse: CTTCCTCTACTTGTGTTCTTC. Induction of somatopause resulted in significant reductions in GHR and IGF-1 expression. Data presented as mean±SEM, tested by 2-way ANOVA, significance accepted at $p<0.05$.
